# Preoperative albumin to carcinoembryonic antigen ratio predicts clinicopathological features and survival in colorectal cancer: a prognostic nomogram study

**DOI:** 10.3389/fonc.2026.1785676

**Published:** 2026-04-14

**Authors:** Yanchun Shi, Shangni Dang, Yan Wang, Ting Sun, Xiaofang Zhang, Weigang Wang

**Affiliations:** Department of Clinical Laboratory, Shanxi Province Cancer Hospital/Shanxi Hospital Affiliated to Cancer Hospital, Chinese Academy of Medical Sciences/Cancer Hospital Affiliated to Shanxi Medical University, Taiyuan, Shanxi, China

**Keywords:** albumin, carcinoembryonic antigen, colorectal cancer, nomogram, prognosis

## Abstract

**Aim:**

This study examined the association between the preoperative albumin-to-carcinoembryonic antigen ratio (ACR) and clinicopathological characteristics in colorectal cancer (CRC) patients and developed and validated a prognostic model incorporating ACR for patient risk stratification.

**Methods:**

A retrospective analysis was performed on the clinical and pathological data of CRC patients who underwent radical surgery at Shanxi Province Cancer Hospital between January 2017 and December 2017. Patient follow-up was conducted, and the Cox proportional hazards model was employed to identify factors influencing overall survival (OS) and disease-free survival (DFS). Additionally, an ACR-based nomogram was developed and its predictive performance was assessed using the concordance index (C-index) and calibration curves. Comparative analyses with the traditional TNM staging system were performed using discriminant indices.

**Results:**

A total of 966 patients with CRC were included in the study, of whom 146 (15.1%) were categorized into the low ACR group and 820 (84.9%) into the high ACR group. The low ACR levels were significantly associated with adverse clinicopathological characteristics and an unfavorable prognosis in patients with CRC. The survival analysis demonstrated that OS (*P* < 0.001) and DFS (*P* < 0.001) were significantly worse in the low ACR group compared to the high ACR group. Multivariate analysis further revealed that high ACR served as an independent protective factor for both OS (HR = 0.433, 95% CI: 0.332 - 0.566; *P* < 0.001) and DFS (HR = 0.545, 95% CI: 0.407- 0.730; *P* < 0.001) among patients with CRC. The ACR-based nomogram demonstrated superior predictive performance, with C-index values of 0.786 for OS and 0.772 for DFS, outperforming the traditional TNM staging system.

**Conclusion:**

Preoperative low ACR is significantly correlated with aggressive tumor characteristics and unfavorable prognosis in CRC patients. The ACR-based nomogram exhibits good predictive accuracy, offering a valuable tool for risk stratification in clinical practice.

## 1 Introduction

Colorectal cancer (CRC) is the third most commonly diagnosed malignancy and the second leading cause of cancer-related mortality worldwide, with approximately 1.9 million new cases and 904,000 deaths reported annually according to GLOBOCAN 2022 (1). Although therapeutic strategies have been gradual improvements, the long-term prognosis for CRC patients remains unsatisfactory, primarily due to the high incidence of postoperative recurrence and distant metastasis (2). Studies indicate that the five-year survival rate for CRC patients was 65% in the United States (3) and 57% in China (4). Therefore, there is an urgent need for reliable prognostic biomarkers that can enhance risk stratification and support the development of individualized treatment plans.

In recent years, peripheral blood parameters have garnered increasing attention in the field of tumor prognosis due to their advantages of being minimally invasive, cost-effective, and suitable for serial monitoring. Numerous studies (5–8) have suggested that combining multiple peripheral blood indicators into composite markers can overcome the limitations of single-parameter models. For instance, composite markers such as the neutrophil-to-lymphocyte ratio (NLR) (5) and albumin-to-globulin ratio (AGR) (8) have shown promising potential in assessing tumor prognosis. Albumin (ALB) is a key biomarker for assessing nutritional status, and malnutrition has been shown to directly compromise the effectiveness of cancer treatment, reduce patients’ quality of life, contribute to organ dysfunction, and increase the risk of complications (9). Consequently, ALB has been widely recognized as an important prognostic indicator across various types of malignancies (9, 10). Carcinoembryonic antigen (CEA), a well-established tumor biomarker, plays a crucial role in the early diagnosis, treatment monitoring, and prognosis assessment of CRC (11). Elevated CEA levels serve as an independent risk factor for both recurrence and metastasis in patients with CRC (12). A progressive increase in CEA levels is often indicative of accelerated disease progression and a poor prognosis.

Composite indices, including the NLR (5), platelet-to-lymphocyte ratio (PLR) (6), C-reactive protein-to-albumin ratio (CAR) (13), and AGR (8), possess prognostic value in oncology. Nevertheless, each of these indices has its own limitations. The NLR and PLR can reflect systemic inflammation, yet they lack tumor - specificity and insights into nutritional status. The CAR combines aspects of inflammation and nutrition, but it depends on C - reactive protein (CRP), which is a non-specific marker. The AGR reflects the nutritional and immune status but overlooks the tumor burden. In contrast, the albumin-to-carcinoembryonic antigen ratio (ACR) uniquely integrates ALB, which is the gold standard for nutritional assessment, and CEA, a highly specific biomarker for CRC. Nevertheless, the prognostic significance of the composite index, ACR, in predicting survival outcomes for CRC patients remains poorly defined and requires further exploration. The ACR integrates information on both the patient’s nutritional status and tumor burden, potentially offering a more comprehensive reflection of disease biology and host compensatory mechanisms. Accordingly, it may represent a promising biomarker for predicting prognosis in cancer patients. Previous studies have constructed nomogram models that incorporated only ACR and TNM staging. In contrast, this study, apart from these two factors, further integrated multiple clinicopathological factors such as vascular invasion, nerves invasion, and ileus, with the aim of enhancing the predictive performance and clinical practical value of the model. This retrospective study, therefore, aimed to investigate the correlation between the ACR and clinicopathological features in CRC patients. Subsequently, a prognostic model incorporating ACR was developed to assess its utility in predicting disease-free survival (DFS) and overall survival (OS), thereby contributing to improved risk stratification in clinical practice.

## 2 Patients and methods

### 2.1 Study population

This retrospective study enrolled patients with CRC who underwent radical resection at Shanxi Province Cancer Hospital between January 2017 and December 2017.

Inclusion criteria:

1. Age ≥ 18 years and pathologically confirmed CRC;
2. Primary CRC managed with radical resection;
3. No prior treatment history for CRC, including surgery, chemotherapy, radiotherapy, or other therapeutic interventions.

Exclusion criteria:

1. Prior treatment received for CRC;
2. History of other malignant diseases;
3. Previous relevant surgeries before enrollment;
4. Discontinuation of treatment following diagnosis.

This study was approved by the Ethics Review Committee of Shanxi Province Cancer Hospital (Approval No. KY2024032). The requirement for informed consent was waived by the ethics committee owing to the retrospective design of the study.

### 2.2 Data collection

The demographic and clinical pathological data of the patients were retrospectively collected by reviewing their medical records. These data included age, gender, history of smoking and alcohol consumption, pathological characteristics (such as TNM stage and presence of nerve or vascular invasion), and preoperative laboratory parameters, specifically ALB and CEA levels. Patients with histologically confirmed CRC were included, and tumor staging was determined according to the 8th edition of the American Joint Committee on Cancer (AJCC) staging system. The ACR was calculated using the following formula: ACR = ALB (g/L)/CEA (μg/L).

### 2.3 Follow-up studies

Follow-up was carried out using a combination of telephone interviews, outpatient consultations, and inpatient examinations, as clinically indicated. OS was defined as the time from the date of surgery to either death from any cause or the last follow-up visit. DFS was calculated as the interval from the date of surgery to the first disease recurrence, death from any cause, or the most recent follow-up. DFS analysis was restricted to patients with stage I–III disease. The final follow-up date was December 31, 2023.

### 2.4 Statistical analyses

Categorical variables were presented as frequencies and percentages, and group comparisons were conducted using the chi-square test or Fisher’s exact test, as appropriate. The “survminer” R package was employed to determine optimal cutoff values, transforming continuous variables into categorical variables. Survival curves were estimated using the Kaplan-Meier method, and intergroup survival differences were evaluated using the log-rank test. Both univariate and multivariate survival analyses were carried out using the Cox proportional hazards regression model. The “survival” and “survminer” R packages were used to generate survival plots. The surv_cutpoint() function in the “survminer” R package determines the optimal cut-off points for continuous variables in survival analysis primarily via the maximally selected rank statistics (Maxstat) method. This approach selects the cut point that maximizes the log-rank statistic between the high and low groups. Restricted cubic spline (RCS) plots were generated using the “plotRCS” R package, while the “rms” R package was utilized for constructing nomograms. The “timeROC” R package was applied to generate time-dependent receiver operating characteristic (ROC) curves, calculate the area under the curve (AUC), and evaluate model performance. All statistical analyses were performed using SPSS (version 27.0) and R (version 4.4.0). A two-sided *P*-value < 0.05 was considered statistically significant.

## 3 Results

### 3.1 Comparison of OS and DFS in patients with CRC across different ACR groups

Based on the inclusion and exclusion criteria, a total of 966 CRC patients were enrolled in this study. The workflow of this study is present in **Supplementary Figure 1**. Using the ‘survminer’ R package, the optimal ACR cutoff value was determined to be 3.5. Accordingly, patients were stratified into two groups: 146 (15.1%) in the low ACR group (< 3.5) and 820 (84.9%) in the high ACR group (≥ 3.5).

**Figure 1:**
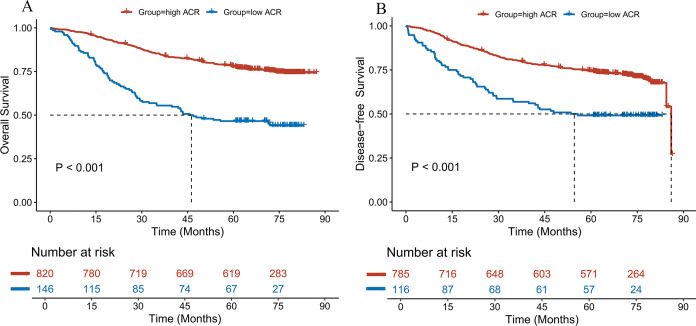
Kaplan-Meier curves comparing **(A)** OS and **(B)** DFS between high- and low-ACR groups.

The 1-, 3-, and 5-year OS rates in the low ACR group were 85.6%, 55.5%, and 46.6%, respectively, whereas the corresponding rates in the high ACR group were 97.0%, 84.9%, and 77.8% (**Supplementary Table 1**). The OS rate was significantly lower in the low ACR group than in the high ACR group (*P* < 0.001; **Figure 1A**). A similar trend was observed for DFS in CRC patients (**Supplementary Table 1**; **Figure 1B**).

**Figure 2:**
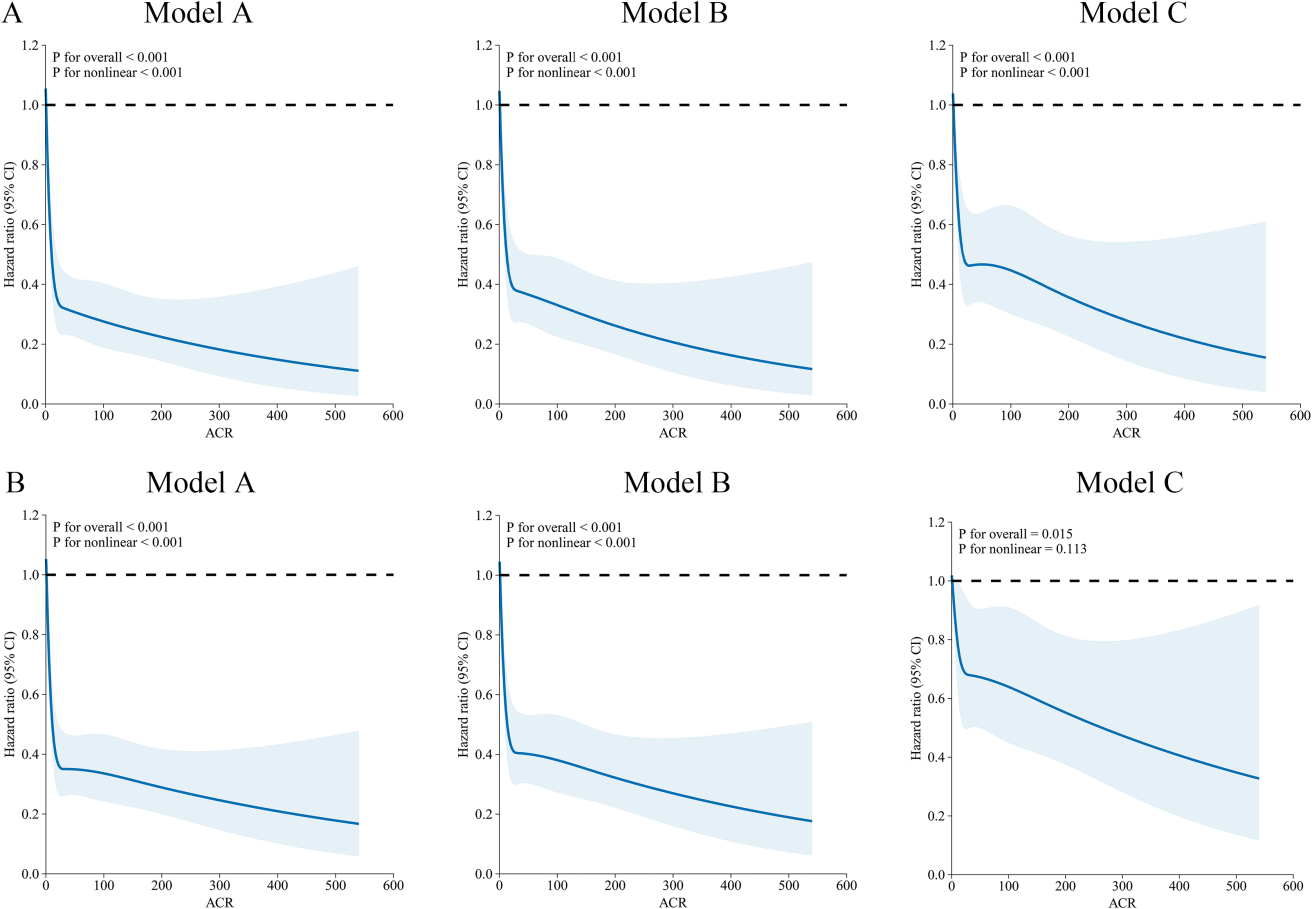
The association between ACR and survival in patients with colorectal Cancer. **(A)** OS, **(B)** DFS. Model A: no adjusted. Model B: adjusted for gender, age, ileus, location, histological type, vascular invasion, and nerves invasion. Model C: adjusted for gender, age, ileus, location, histological type, vascular invasion, nerves invasion, T stage, N stage, and M stage.

Further analysis revealed a nonlinear association between preoperative ACR levels and patient prognosis. RCS analysis demonstrated an L-shaped relationship between ACR levels and the risk of OS/DFS, indicating that higher ACR levels were linked to a progressively lower risk of both mortality and tumor recurrence. Importantly, this inverse association remained statistically significant after adjusting for multiple potential confounding factors (**Figures 2A, B**).

### 3.2 Association between ACR and clinicopathological characteristics

A comparison of clinicopathological characteristics between the low ACR and high ACR groups indicated statistically significant differences in the presence or absence of ileus (*χ²* = 24.057, *P* < 0.001), tumor location (*χ²* = 10.136, *P* = 0.001), vascular invasion (*χ²* = 5.220, *P* = 0.022), T stage (*χ²* = 22.266, *P* < 0.001), N stage (*χ²* = 19.952, *P* < 0.001), M stage (*χ²* = 52.335, *P* < 0.001), and TNM stage (*χ²* = 70.301, *P* < 0.001). No significant differences were observed between the two groups in p53 mutation status or microsatellite instability (MSI) status (both *P* > 0.05). These findings suggest that patients in the low ACR group presented with more aggressive tumor characteristics and significantly poorer clinical and pathological outcomes compared to those in the high ACR group (**Table 1**).

**Table 1:** The correlation between ACR and the clinicopathological features of CRC patients (n, %).

Characteristics	Low ACR (n = 146)	High ACR (n = 820)	χ²	*P*
Gender			3.273	0.070
Male	73 (50.0)	476 (58.0)		
Female	73 (50.0)	344 (42.0)		
Age			0.049	0.825
≤ 60 yr	70 (47.9)	385 (47.0)		
> 60 yr	76 (52.1)	435 (53.0)		
Ileus			24.057	< 0.001
Yes	54 (37.0)	152 (18.5)		
No	92 (63.0)	668 (81.5)		
Location			10.136	0.001
Colon	86 (58.9)	366 (44.6)		
Rectum	60 (41.1)	454 (55.4)		
Histological type			0.353	0.553
Ulcerative type	125 (85.6)	686 (83.7)		
Non-ulcerative type	21 (14.4)	134 (16.3)		
Vascular invasion			5.220	0.022
Yes	31 (21.2)	114 (13.9)		
No	115 (78.8)	706 (86.1)		
Nerves invasion			1.137	0.286
Yes	14 (9.6)	58 (7.1)		
No	132 (90.4)	762 (92.9)		
T stage			22.266	< 0.001
T1	0 (0)	21 (2.6)		
T2	12 (8.2)	180 (22.0)		
T3	58 (39.7)	312 (38.0)		
T4	76 (52.1)	307 (37.4)		
N stage			19.952	< 0.001
N0	61 (41.8)	494 (60.2)		
N1	40 (27.4)	182 (22.2)		
N2	45 (30.8)	144 (17.6)		
M stage			52.335	< 0.001
M0	116 (79.5)	785 (95.7)		
M1	30 (20.5)	35 (4.3)		
TNM stage			70.301	< 0.001
I	6 (4.1)	167 (20.3)		
II	45 (30.8)	310 (37.8)		
III	65 (44.5)	308 (37.6)		
IV	30 (20.6)	35 (4.3)		
p53 (n = 733)			1.860	0.173
wild−type	92 (78.0)	442 (71.9)		
mutant	26 (22.0)	173 (28.1)		
MSI-H (n = 734)			0.357	0.464
Yes	4 (4.2)	39 (6.1)		
No	91 (95.8)	600 (93.9)		
Disease progression at last follow-up			44.218	< 0.001
Yes	89 (61.0)	264 (32.2)		
No	57 (39.0)	556 (67.8)		
Status at last follow-up			55.097	< 0.001
Dead	80 (54.8)	201 (24.5)		
Censored	66 (45.2)	619 (75.5)		

**Figure 3:**
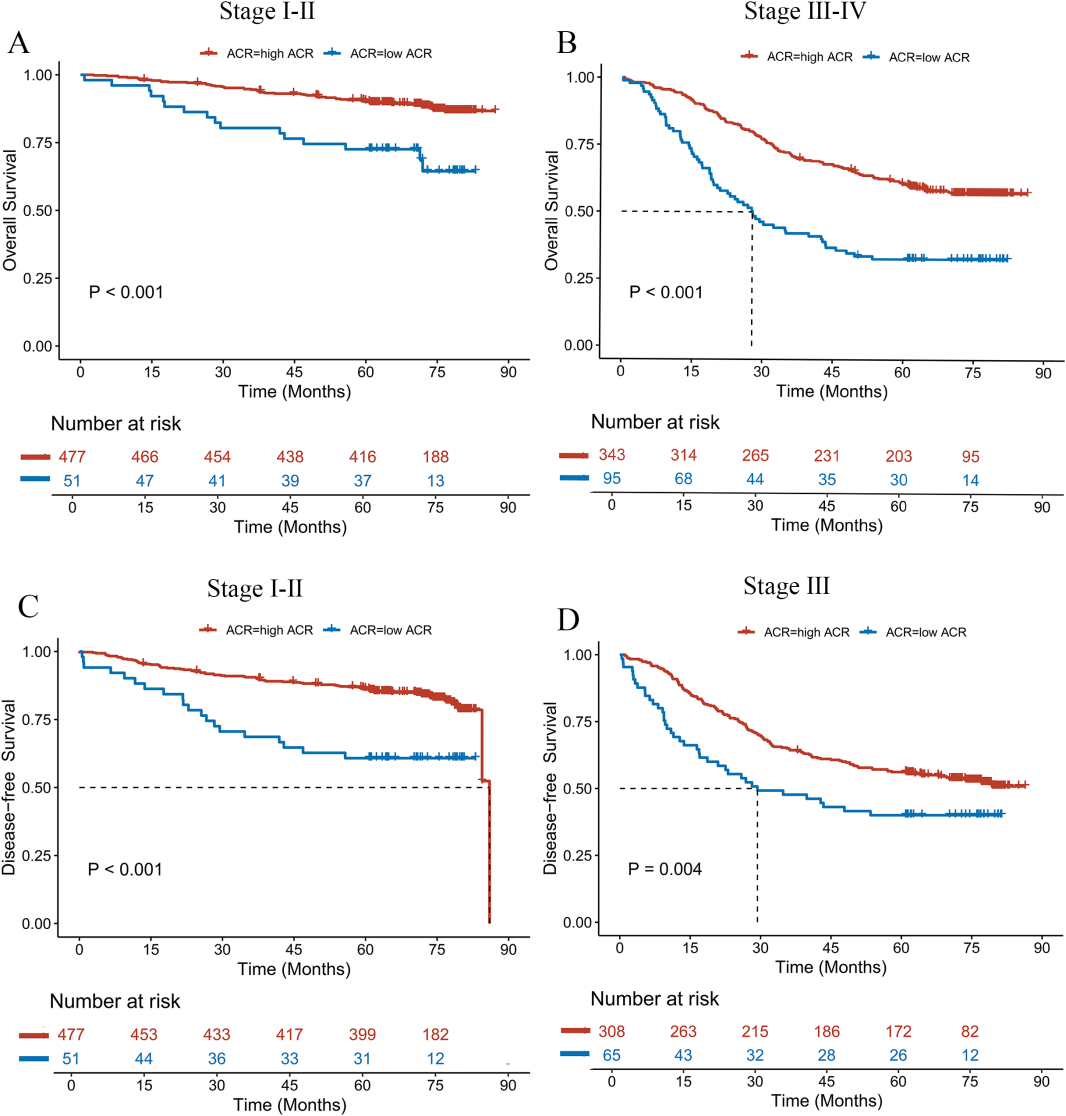
Kaplan-Meier survival curves for **(A, B)** OS and **(C, D)** DFS stratified by ACR levels across different TNM stages.

CRC patients were categorized into early-stage (I–II) and advanced-stage (III–IV) groups according to the TNM staging system. Differences in OS and DFS between the high ACR and low ACR groups across stages were analyzed. The results indicated that patients in the high ACR group exhibited significantly better prognoses compared to those in the low ACR group, irrespective of disease stage (all *P* < 0.05, **Figures 3A–D**). Using the same stratification approach, patients were stratified according to T stage, N stage, and M stage. The differences in OS among patients with varying ACR levels within each subgroup were then analyzed. The results indicated that, with the exception of patients in the T1-T2 stages — where no significant differences in OS were observed across ACR levels — the OS of the low ACR group was significantly poorer compared to that of the high ACR group in all other subgroups (**Supplementary Figures 2A**–**F**). A similar trend was also evident in the DFS analysis (**Supplementary Figures 3A**–**E**).

To evaluate potential confounding effects, we conducted multivariable subgroup analyses across various clinical characteristics, which consistently identified ACR as an independent predictor of both OS and DFS across most subgroups (**Supplementary Figures 4A, B**). The Sankey diagram illustrates that within the low ACR group, 20.55% (30/146) of patients were at stage IV, of which 93.33% (28/30) ultimately succumbed to the disease. Additionally, 44.52% (65/146) were classified as stage III, with a mortality rate of 55.38% (36/65). In comparison, in the high ACR group, only 4.27% (35/820) were at stage IV, and 37.56% (308/820) were at stage III, with mortality rates of 77.14% (27/35) and 37.99% (117/308), respectively (**Supplementary Figure 5A**). The DFS analysis also demonstrated a fully consistent trend (**Supplementary Figure 5B**).

The aforementioned results indicate that low ACR is not only closely correlated with more aggressive clinicopathological characteristics, but also directly linked to unfavorable survival outcomes in CRC patients.

### 3.3 Univariate and multivariate analysis of OS and DFS

The results of multivariate Cox regression analysis demonstrated that age (HR = 2.006, 95% CI: 1.565-2.572, *P* < 0.001), ileus (HR = 1.489, 95% CI: 1.143-1.940, *P* = 0.003), vascular invasion (HR = 1.802, 95% CI: 1.339-2.427, *P* < 0.001), nerves invasion (HR = 1.917, 95% CI: 1.348-2.726, *P* < 0.001), N stage (HR = 2.459, 95% CI: 1.854-3.260, *P* < 0.001), M stage (HR = 4.214, 95% CI: 3.060-5.805, *P* < 0.001), and ACR level (HR = 0.433, 95% CI: 0.332-0.566, *P* < 0.001) were independent prognostic factors influencing OS in patients with CRC (**Table 2**). Importantly, the ACR level (HR = 0.545, 95% CI: 0.407-0.730, *P* < 0.001) was also significantly associated with DFS (**Table 3**).

**Table 2:** Univariate and multivariate analysis of OS in CRC patients.

Characteristics	Univariate analysis		Multivariate analysis	
	HR (95%CI)	*P*	HR (95%CI)	*P*
Gender				
Female	Ref.			
Male	1.077 (0.849-1.365)	0.543		
Age				
≤ 60 yr	Ref.		Ref.	
> 60 yr	1.706 (1.338-2.176)	< 0.001	2.006 (1.565-2.572)	< 0.001
Smoking				
No	Ref.			
Yes	0.927 (0.719-1.196)	0.562		
Drinking				
No	Ref.			
Yes	0.797 (0.569-1.118)	0.190		
Hypertension				
No	Ref.			
Yes	1.094 (0.851-1.406)	0.485		
Diabetes				
No	Ref.			
Yes	1.179 (0.838-1.660)	0.344		
Location				
Rectum	Ref.		Ref.	
Colon	1.226 (1.002-1.600)	0.048	1.042 (0.813-1.335)	0.746
Ileus				
No	Ref.		Ref.	
Yes	2.202 (1.716-2.827)	< 0.001	1.489 (1.143-1.940)	0.003
Vascular invasion				
No	Ref.		Ref.	
Yes	3.998 (3.109-5.141)	< 0.001	1.802 (1.339-2.427)	< 0.001
Nerves invasion				
No	Ref.		Ref.	
Yes	3.594 (2.631-4.909)	< 0.001	1.917 (1.348-2.726)	< 0.001
Histological type				
Non-ulcerative type	Ref.		Ref.	
Ulcerative type	1.508 (1.049-2.168)	0.027	1.128 (0.772-1.649)	0.534
T stage				
T1-T2	Ref.		Ref.	
T3-T4	2.970 (2.010-4.389)	< 0.001	1.335 (0.868-2.053)	0.188
N stage				
N0	Ref.		Ref.	
N1-N2	3.814 (2.960-4.915)	< 0.001	2.459 (1.854-3.260)	< 0.001
M stage				
M0	Ref.		Ref.	
M1	7.445 (5.520-10.041)	< 0.001	4.214 (3.060-5.805)	< 0.001
ACR				
Low	Ref.		Ref.	
High	0.325 (0.251-0.421)	< 0.001	0.433 (0.332-0.566)	< 0.001

**Table 3:** Univariate and multivariate analysis of DFS in CRC patients.

Characteristics	Univariate analysis		Multivariate analysis	
	HR (95%CI)	*P*	HR (95%CI)	*P*
Gender				
Female	Ref.			
Male	1.114 (0.880-1.410)	0.368		
Age				
≤ 60 yr	Ref.		Ref.	
> 60 yr	1.519 (1.197-1.927)	0.001	1.477 (1.163-1.876)	0.001
Smoking				
No	Ref.			
Yes	1.062 (0.830-1.358)	0.663		
Drinking				
No	Ref.			
Yes	0.982 (0.720-1.338)	0.906		
Hypertension				
No	Ref.			
Yes	0.922 (0.714-1.190)	0.532		
Diabetes				
No	Ref.			
Yes	1.087 (0.772-1.530)	0.344		
Location				
Rectum	Ref.			
Colon	1.031 (0.817-1.302)	0.796		
Ileus				
No	Ref.		Ref.	
Yes	1.533 (1.170-2.008)	0.002	1.223 (0.926-1.616)	0.156
Vascular invasion				
No	Ref.		Ref.	
Yes	3.270 (2.504-4.271)	< 0.001	1.949 (1.446-2.628)	< 0.001
Nerves invasion				
No	Ref.		Ref.	
Yes	2.943(2.090-4.144)	< 0.001	1.854 (1.285-2.676)	< 0.001
Histological type				
Non-ulcerative type	Ref.		Ref.	
Ulcerative type	1.823 (1.242-2.676)	0.002	1.450 (0.973-2.161)	0.068
T stage				
T1-T2	Ref.		Ref.	
T3-T4	2.199 (1.574-3.072)	< 0.001	1.257 (0.877-1.802)	0.213
N stage				
N0	Ref.		Ref.	
N1-N2	3.103 (2.443-3.942)	< 0.001	2.265 (1.747-2.936)	< 0.001
ACR				
Low	Ref.		Ref.	
High	0.438 (0.329-0.583)	< 0.001	0.545 (0.407-0.730)	< 0.001

### 3.4 Validation of the relationship between ACR and both OS and DFS

To further assess the clinical utility of ACR in patients with CRC, we randomly divided the 966 cases into a training cohort (n = 677) and a validation cohort (n = 289) at a 7:3 ratio. The clinicopathological features of CRC patients in both cohorts are summarized in **Supplementary Table 2**. No statistically significant differences were identified in baseline characteristics between the two cohorts, thereby confirming their comparability for subsequent validation analyses. To verify the reasonableness of setting the ACR cutoff value at 3.5, we calculated the optimal cutoff values in the training set and validation set, which were 7.7 and 3.3, respectively. Subsequently, we compared the C-indexes of the OS and DFS nomogram models based on ACR at the three different cutoff values of 3.5, 7.7, and 3.3. The results indicated that the model exhibited the best performance when the cutoff value was 3.5 (**Supplementary Table 3**).

**Figure 4:**
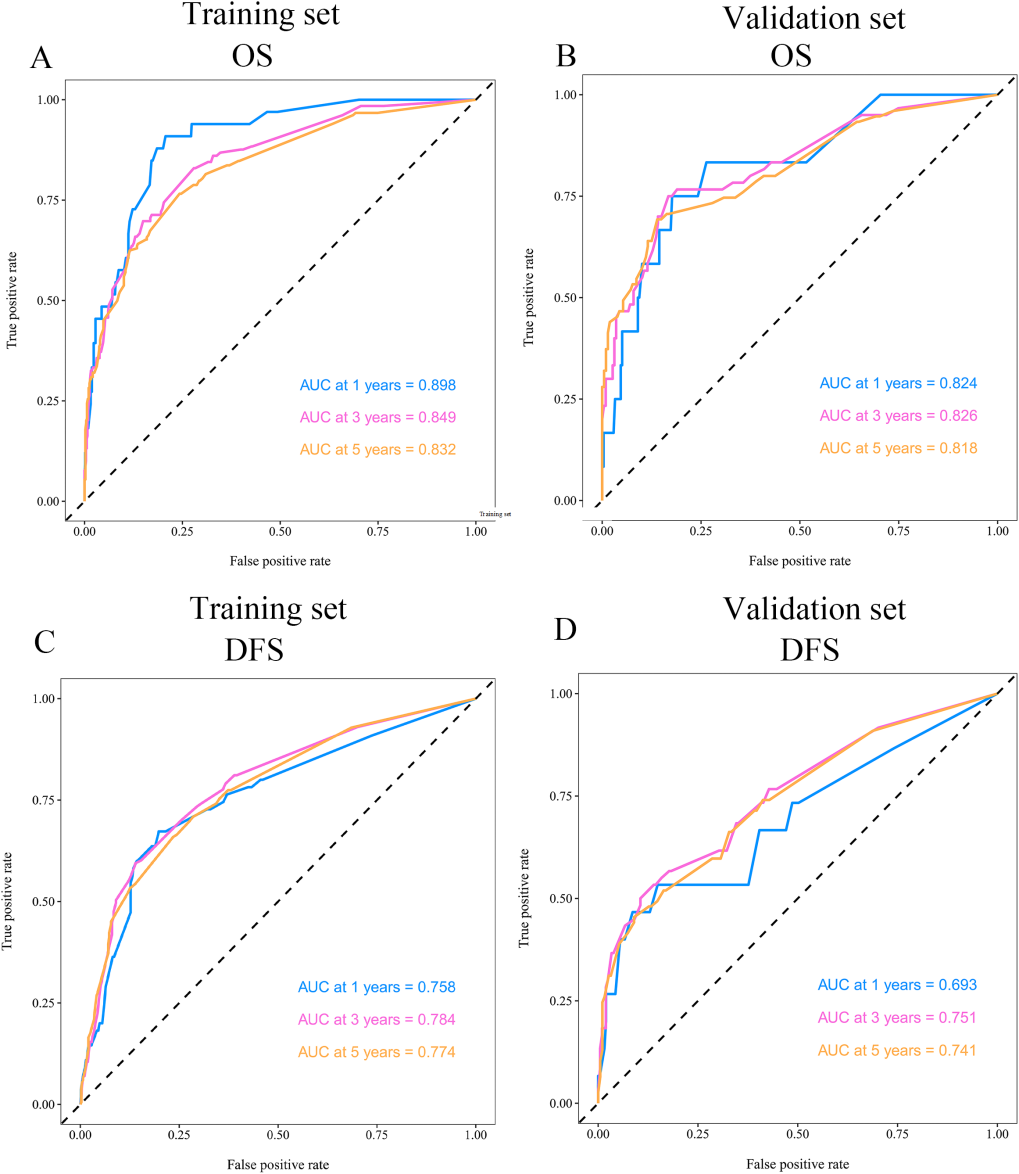
Time-dependent ROC curves assessing the predictive performance of the model for **(A, B)** OS and **(C, D)** DFS in the training and validation sets.

The predictive performance of 1-year, 3-year, and 5-year OS and DFS was evaluated in both the training and validation cohorts using time-dependent ROC curve analysis, and the corresponding AUC values were calculated. The results indicated that in the training cohort, the AUC values for OS prediction at 1-year, 3-year, and 5-year were 0.898, 0.849, and 0.832, respectively (**Figure 4A**), while in the validation cohort, the corresponding AUC values were 0.824, 0.826, and 0.818 (**Figure 4B**). Regarding DFS prediction, the AUC values in the training cohort at 1-year, 3-year, and 5-year were 0.758, 0.784, and 0.774, respectively (**Figure 4C**), whereas in the validation cohort, they were 0.693, 0.751, and 0.741, respectively (**Figure 4D**). Calibration curves demonstrated good agreement between predicted and observed probabilities for 3- and 5-year OS (**Supplementary Figures 6A**–**D**) and DFS (**Supplementary Figures 7A**–**D**), further supporting the model’s clinical applicability.

### 3.5 Development of an ACR-incorporated nomogram

**Figure 5:**
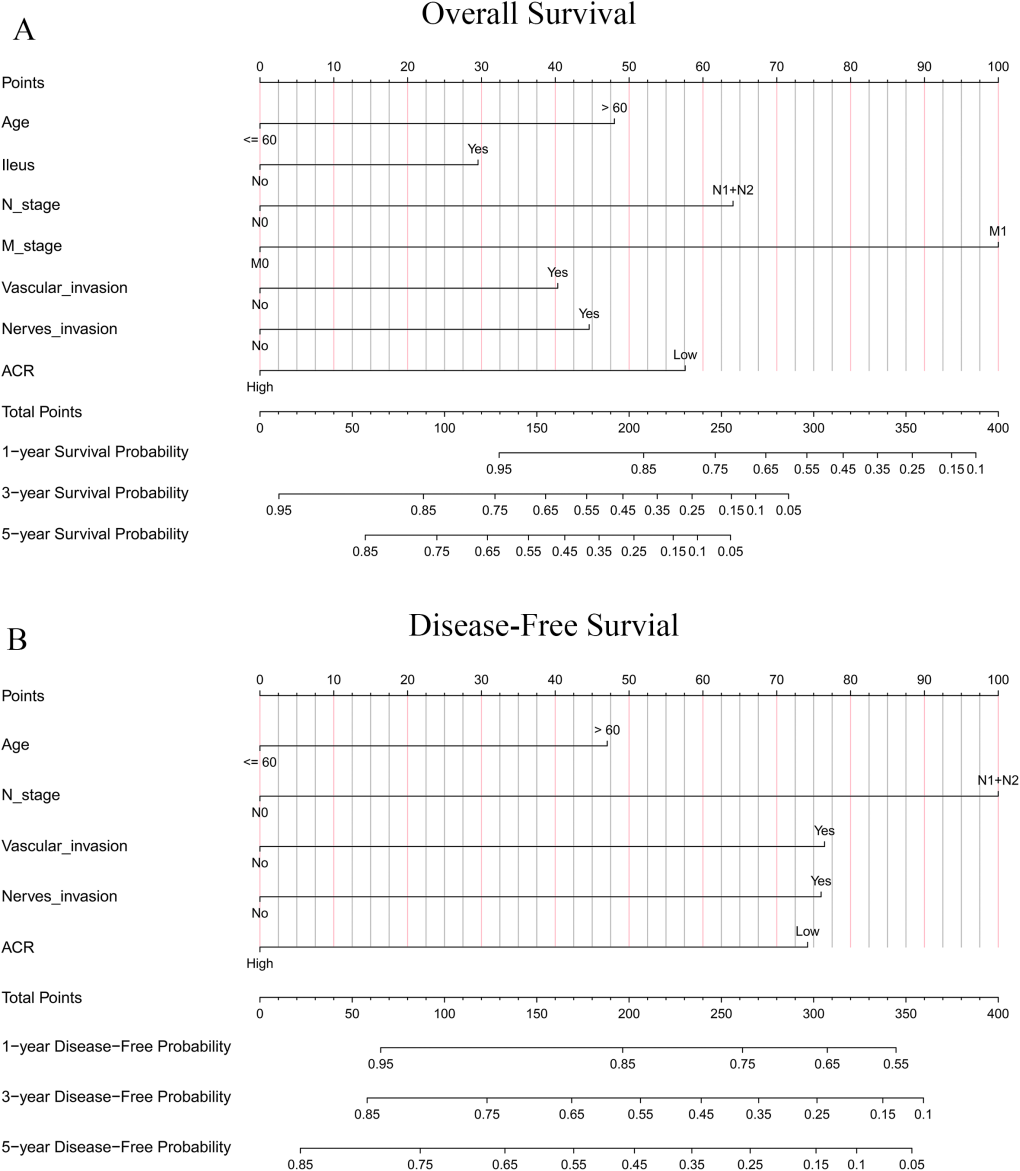
Prognostic nomograms for **(A)** OS and **(B)** DFS incorporating ACR and other clinical predictors.

Based on the independent prognostic factors identified through multivariate Cox regression analysis, we developed two clinical prediction models. To verify whether the nomogram constructed based on ACR is superior to those constructed based on CEA or ALB, we compared three nomograms built on the same baseline variables but incorporating ACR, CEA, and ALB respectively. The results showed that the nomogram incorporating ACR performed slightly better than the other two (**Supplementary Table 4**). **Figure 5A** illustrates the ACR-incorporated nomogram for predicting OS in CRC patients, while **Figure 5B** displays the ACR-incorporated nomogram for predicting DFS, with the C-indexes of 0.786 and 0.722, respectively (**Table 4**).

**Table 4:** Comparison of discriminatory ability between nomograms and TNM stage for CRC patients.

Models	C-index			NRI		IDI	
	Value	Difference	*P*	Difference	*P*	Difference	*P*
OS							
TNM stage	0.751	Ref.		Ref.		Ref.	
OS nomogram	0.786	0.036(0.018-0.056)	< 0.001	0.210 (0.121-0.302)	< 0.001	0.066 (0.035-0.103)	< 0.001
DFS							
TNM stage	0.693	Ref.		Ref.		Ref.	
DFS nomogram	0.722	0.029 (0.010-0.051)	0.003	0.171 (0.005 – 0.246)	0.044	0.038 (0.011- 0.071)	0.014

Using the ACR-incorporated nomogram scoring system, we calculated the total points for all patients and determined the optimal prognostic cutoff values for OS and DFS using the “survminer” R package. The cutoff values for OS and DFS were 128 and 174, respectively. Using these thresholds, patients were categorized into high-risk groups (total points ≥ cutoff value) and low-risk groups (total points < cutoff value). Kaplan-Meier survival analysis demonstrated that both OS (**Supplementary Figure 8A**, *P* < 0.001) and DFS (**Supplementary Figure 8B**, *P* < 0.001) were significantly lower in the high-risk group compared to the low-risk group. These findings confirm that the nomogram prediction model incorporating ACR exhibits strong prognostic stratification capability and can effectively identify colorectal cancer patients at high risk of poor prognosis.

### 3.6 Comparative analysis of the ACR-incorporated nomogram and the TNM staging system

**Figure 6:**
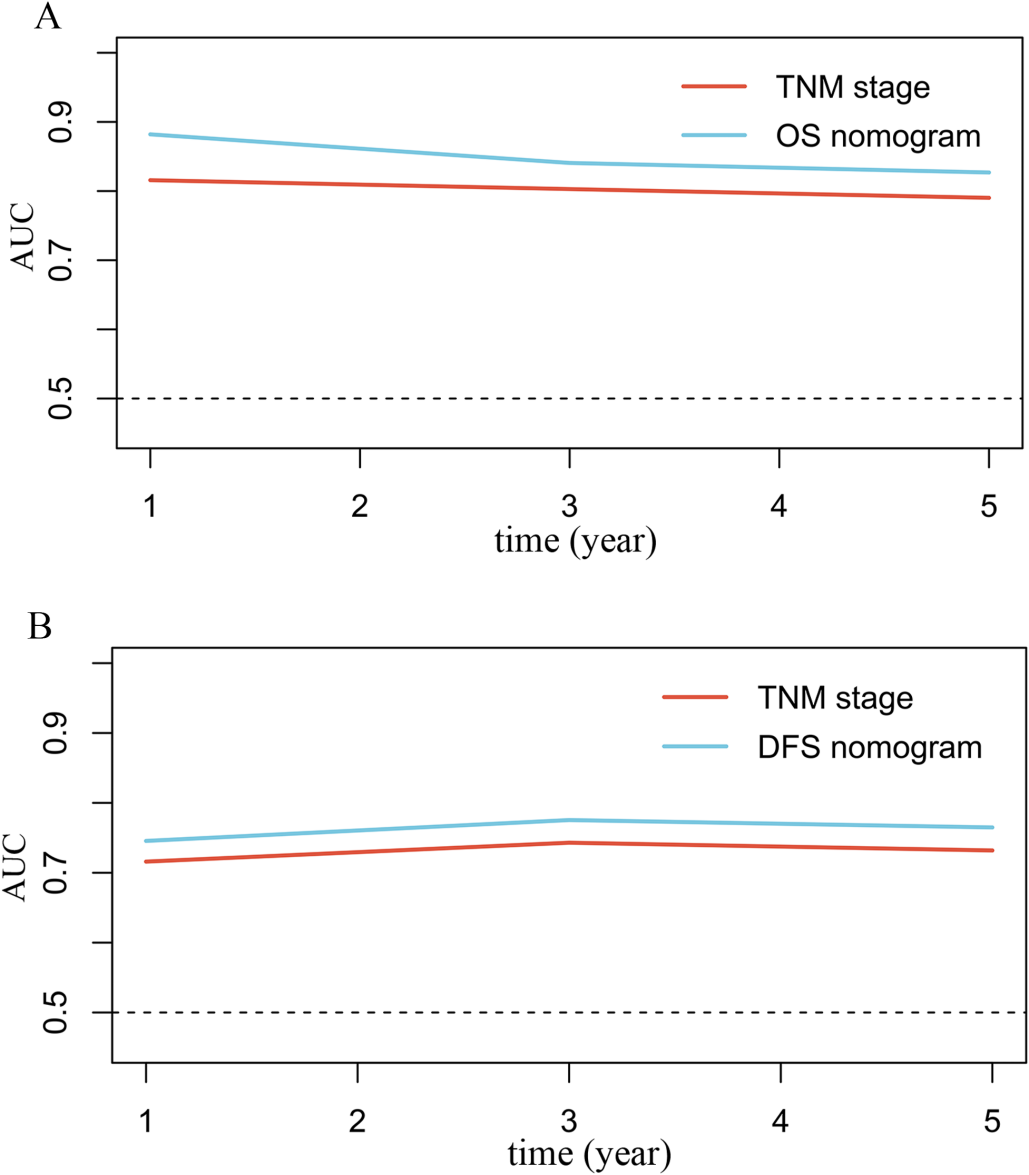
Time-dependent ROC curve analysis comparing the prognostic performance of the nomogram and the conventional TNM staging system for OS **(A)** and DFS **(B)**.

Time-dependent ROC curve analysis indicated that the nomogram exhibited superior prognostic performance compared to the conventional TNM staging system in predicting both OS (**Figure 6A**) and DFS (**Figure 6B**) in CRC patients. Quantitative evaluation using the C-index, net reclassification improvement (NRI), and integrated discrimination improvement (IDI) further confirmed statistically significant improvements in prognostic accuracy when using the nomogram. Specifically, the OS nomogram demonstrated a 3.6% increase in C-index, a 21.0% improvement in NRI, and a 6.6% increase in IDI compared to TNM staging (all *P* < 0.001; **Table 4**). Similarly, the DFS nomogram showed improvements of 2.9% in C-index, 17.1% in NRI, and 3.8% in IDI relative to TNM staging (all *P* < 0.05; **Table 4**).

## 4 Discussion

Despite advances in CRC diagnosis and treatment, long-term survival remains suboptimal, highlighting the need for better prognostic tools. This study found that preoperative low ACR correlates with poor clinicopathological features and worse outcomes. We developed an ACR-based prognostic model with strong predictive and risk-stratification capabilities and is conducive to the formulation of follow - up strategies.

Serum ALB level serves as a crucial biomarker for evaluating nutritional status. Hypoalbuminemia not only compromises immune function and reduces the effectiveness of antitumor therapies (14, 15), but is also strongly associated with systemic inflammatory responses (16, 17). Accumulating evidence suggests that inflammatory cytokines, such as interleukin-6, can inhibit albumin synthesis in hepatocytes, thereby lowering serum ALB levels (18). Consequently, preoperative serum ALB levels may function as a comprehensive indicator reflecting tumor prognosis, inflammatory activity, and overall nutritional status. In light of these findings, several ALB-based composite indices have been developed and applied for the prediction of prognosis in CRC patients (14, 19, 20). *Zhen* et al. showed that preoperative D−dimer to albumin ratio (DAR) was a significant predictor of outcomes in locally advanced rectal cancer, independently associated with OS (HR = 1.941) and DFS (HR = 1.715). Their DAR-based nomogram demonstrated predictive accuracy (OS: C-index = 0.743; DFS: C-index = 0.705) (14). Notably, the nomogram developed in this study shows modestly improved predictive performance, with C-indexes reaching 0.786 for OS and 0.722 for DFS. This difference can be ascribed to the fact that DAR integrates coagulation function with nutritional and inflammatory status. In contrast, ACR encompasses nutritional and inflammatory status as well as tumor burden, thereby offering a more comprehensive portrayal of the host - tumor interaction. Additionally, the nomogram model developed in this study incorporated significant pathological features, which also contributed to enhancing its predictive performance. A meta-analysis demonstrated that CAR serves as an independent predictor of poor OS (HR = 2.25; 95% CI: 1.84-2.76) and DFS (HR = 2.49; 95% CI: 1.43-4.33) in patients with CRC (21). Likewise, *Li* et al. demonstrated that AGR may serve as a reliable prognostic indicator for CRC patients (8). *Miyata* et al. showed that elevated albumin-total lymphocyte count-RAS index (ALRI) predicts worse outcomes in resectable CRC, with high-ALRI patients exhibiting lower 5-year recurrence-free survival (RFS, 59.4% *vs.* 81.1%) and OS (75.3% *vs.* 93.2%) compared to those in the low-ALRI group (22). However, the high cost associated with RAS mutation testing may hinder its widespread clinical implementation (23). In addition, due to the limited sample size for detecting BRAF, K-RAS, and N-RAS mutations in this study, these genetic alterations were not included in the analysis, which warrants further investigation in future studies.

Meanwhile, CEA, a well-established biomarker in CRC diagnosis and management, has been recognized as an independent prognostic factor for CRC based on preoperative measurements. Furthermore, dynamic postoperative monitoring of CEA levels, particularly when exceeding 5 ng/mL, enables early prediction of recurrence risk and demonstrates a significant association with cancer-specific mortality and reduced OS, as confirmed by multiple studies. Certainly, the diagnostic and prognostic utility of CEA as a standalone biomarker for CRC is limited. Consequently, integrating CEA with other biomarkers can improve the overall accuracy and effectiveness of diagnosis and prognosis. *Cai* et al. found that preoperative CEA and the systemic inflammation response index (C-SIRI, HR = 2.563) were independent predictors of OS in patients with CRC (24). Additionally, incorporating dynamic longitudinal data of postoperative CEA and other tumor markers can significantly improve the predictive accuracy of prognostic models. Evidently, the changes in dynamic monitoring indicators are of great significance for improving the model’s performance. Therefore, subsequent research should conduct in-depth tracking of the dynamic evolution of the ACR to continuously optimize the model’s predictive efficiency.

As a composite index consisting of ALB and CEA, the ACR integrates critical information regarding the host’s nutritional status, inflammatory response, and tumor burden, thereby offering comprehensive value in the prognostic assessment of CRC patients. *Xie* et al. demonstrated that low ACR levels were significantly correlated with increased tumor invasiveness and an unfavorable prognosis in patients with rectal cancer. Moreover, ACR was identified as an independent prognostic factor for both progression-free survival (PFS, HR = 0.581) and OS (HR = 0.560) (19), a result consistent with the findings of the present study. Nevertheless, the slightly higher C-index observed in our model (OS: 0.786 *vs.* 0.699) may be explained by the integration of ACR with other clinically relevant factors, including vascular invasion, nerves invasion, and ileus, which collectively improved predictive performance. Although the TNM staging system is widely utilized for prognostic assessment in CRC, considerable prognostic heterogeneity persists among patients classified under the same stage. Our study demonstrates that the ACR-based model exhibits improved predictive accuracy compared to the TNM staging system, highlighting its potential as a complementary biomarker for achieving more precise prognostic stratification of CRC patients. Additionally, the measurement of serum ALB and CEA is routinely performed in clinical settings due to its cost-effectiveness and ease of implementation, suggesting considerable potential for widespread application. It is important to emphasize that, while the ACR-based nomogram demonstrated a moderately enhanced prognostic performance when compared with albumin alone, CEA alone, or previously reported albumin-related indices, the extent of this improvement was relatively limited. Consequently, the superiority of the ACR model should be interpreted with caution. Instead of substituting conventional biomarkers or the TNM staging system, the ACR-based model functions as a supplementary tool to optimize risk stratification and support individualized clinical decision-making.

However, this study has several limitations that should be acknowledged. First, the retrospective nature and single-center design may introduce inherent selection bias. Second, the dynamic changes in ALB and CEA levels during treatment and follow-up were not fully integrated into the analysis, which could potentially influence the robustness of the findings. Third, the absence of external validation restricts the generalizability of the results. Fourth, the optimal cutoff value for ACR identified in this study by means of maximally selected rank statistics is inherently dataset - dependent. This approach, although valuable for exploratory analysis, has a tendency to produce thresholds that might not be directly applicable to other populations. As a result, the generalizability of this particular cutoff is restricted, and it requires further validation via larger, multicenter studies with diverse external datasets to ascertain its stability and clinical utility. Therefore, future multicenter, prospective studies are necessary to further validate these conclusions.

## 5 Conclusion

This study revealed that preoperative low ACR levels were significantly associated with adverse clinicopathological characteristics and an unfavorable prognosis in patients with CRC. The ACR-based nomogram demonstrated superior discriminatory performance when compared to the traditional TNM staging system, which allows for a precise individualized assessment of the risk of recurrence and mortality. This model is a valuable tool for clinical risk stratification and the development of subsequent follow-up strategies.

## Data Availability

The raw data supporting the conclusions of this article will be made available by the authors, without undue reservation.
