# Supplementary material for: Preoperative albumin to carcinoembryonic antigen ratio predicts clinicopathological features and survival in colorectal cancer: a prognostic nomogram study
Source: Front Oncol. 2026 Apr 14;16:1785676. doi: 10.3389/fonc.2026.1785676 (PMC13121080; doi:10.3389/fonc.2026.1785676)
Supplement: Supplementary file 1 [file DataSheet1.docx]

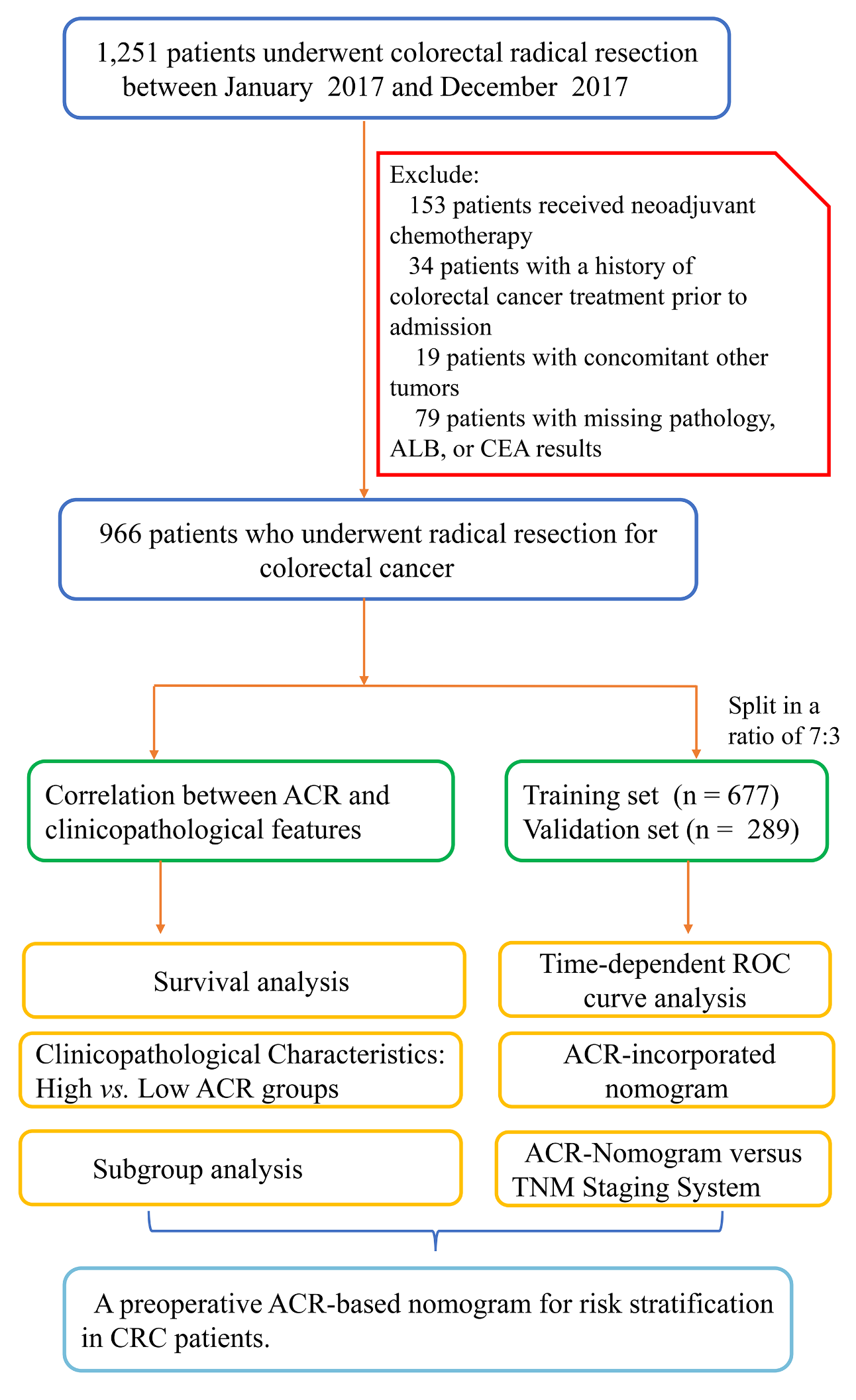


**Figure S1.** Workflow of this study


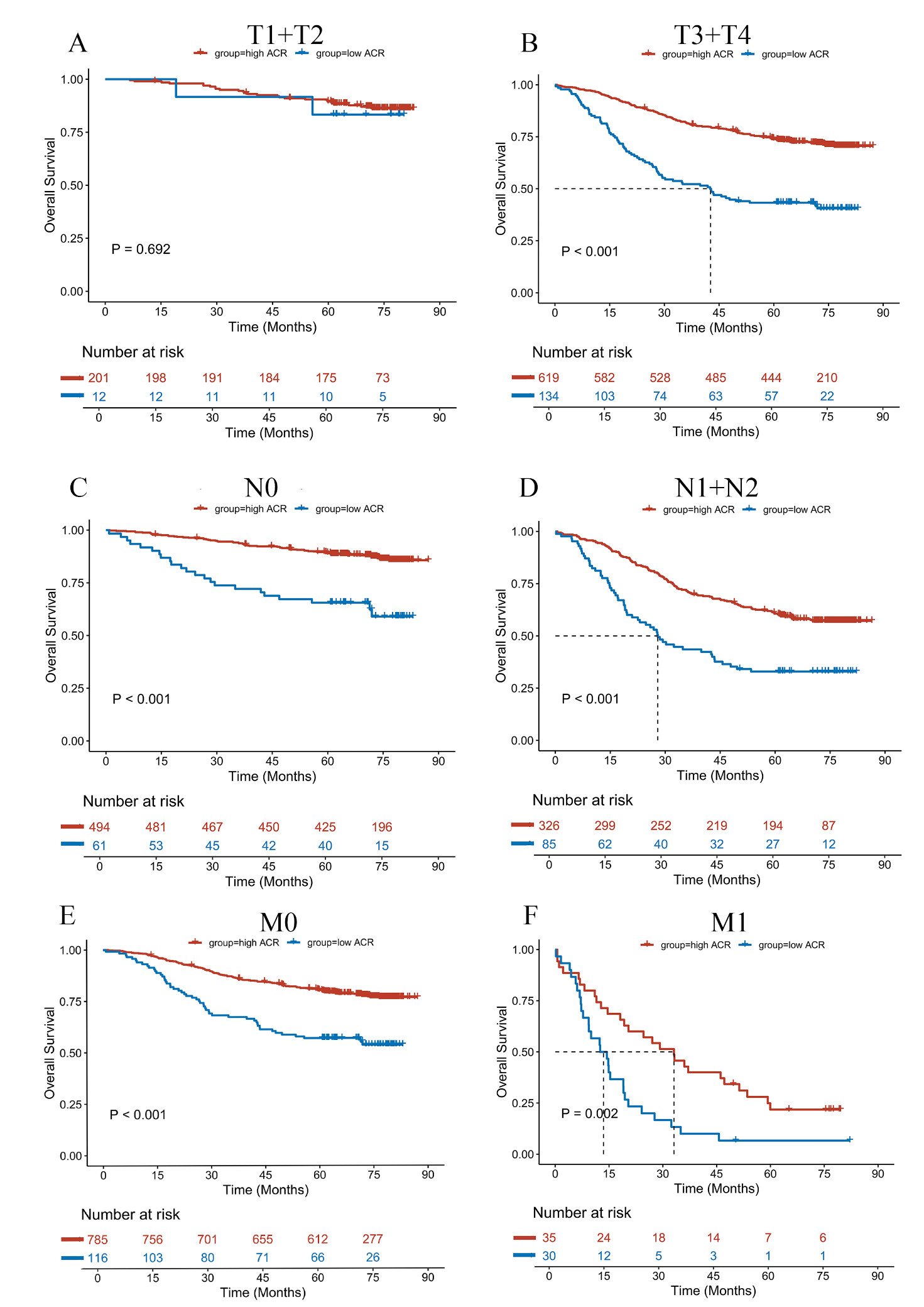


**Figure S2.** Overall Survival (OS) Stratified by ACR Levels Across Different T stages (A, B), N stages (C, D), and M stages (E, F).


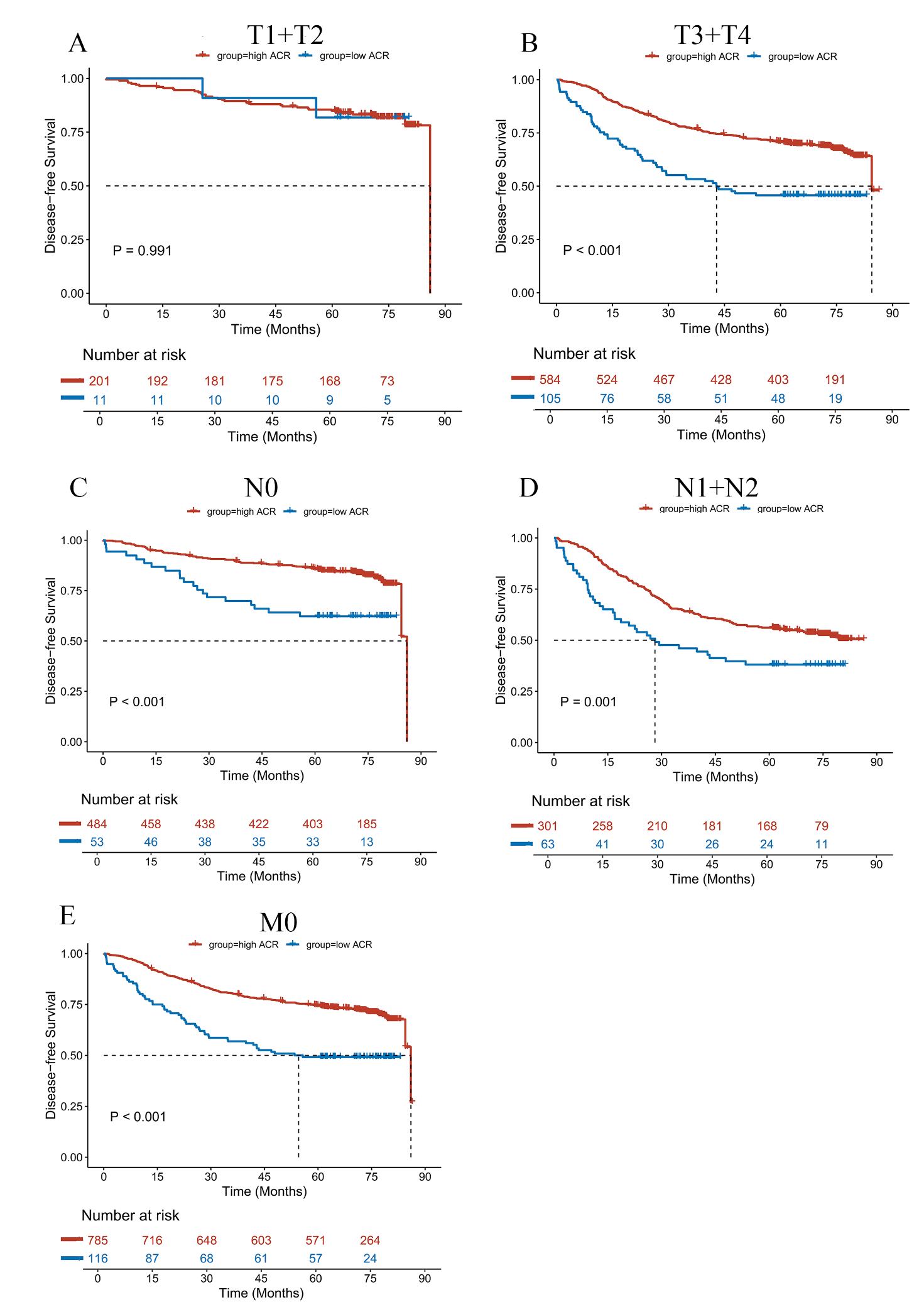


**Figure S3.** Disease-free survival (DFS) Stratified by ACR Levels Across Different T stages (A, B), N stages (C, D), and M stage (E).


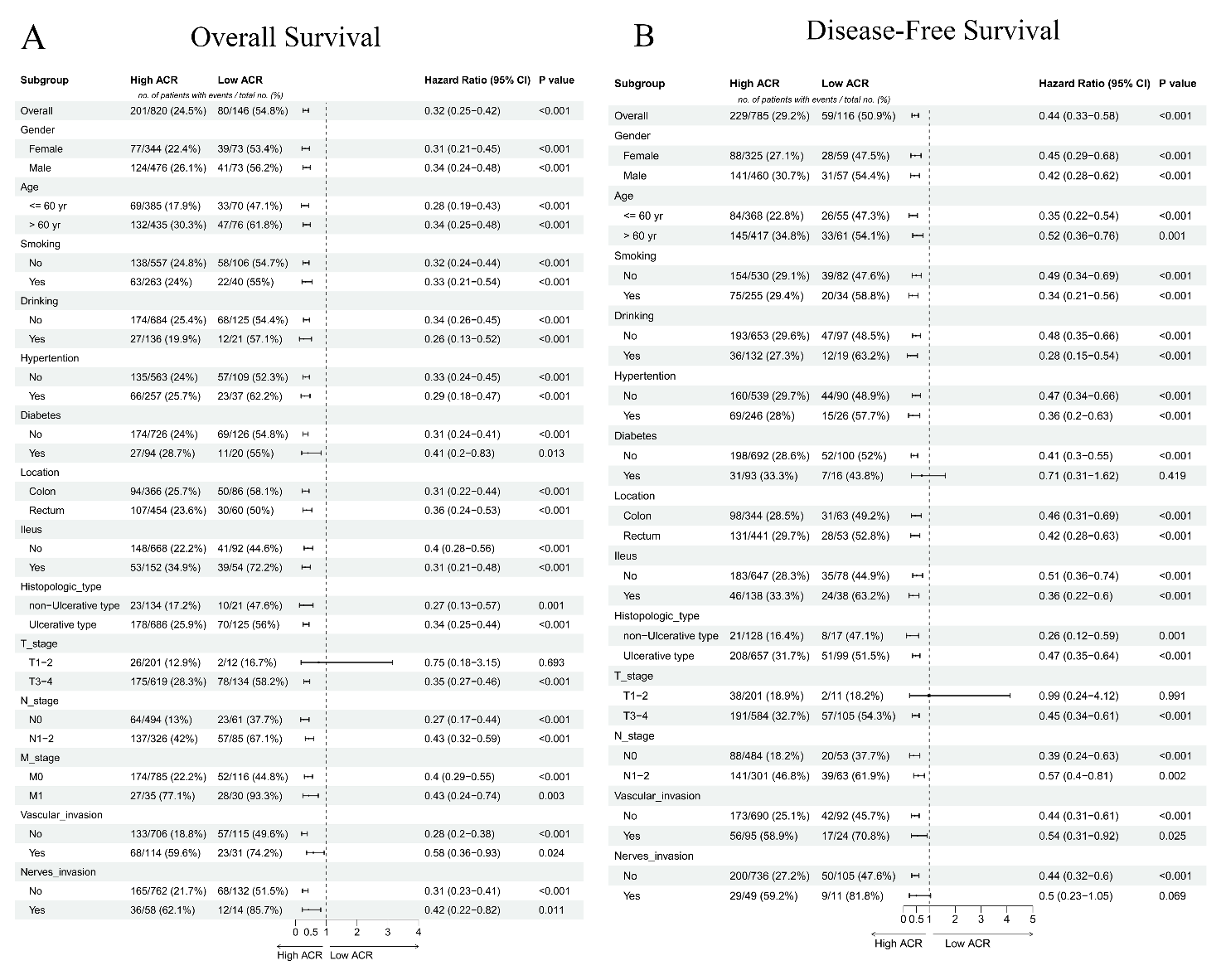


**Figure S4.** The association between ACR and hazard risk of OS and DFS in various subgroups. (A, overall survival, B, disease-free survival).


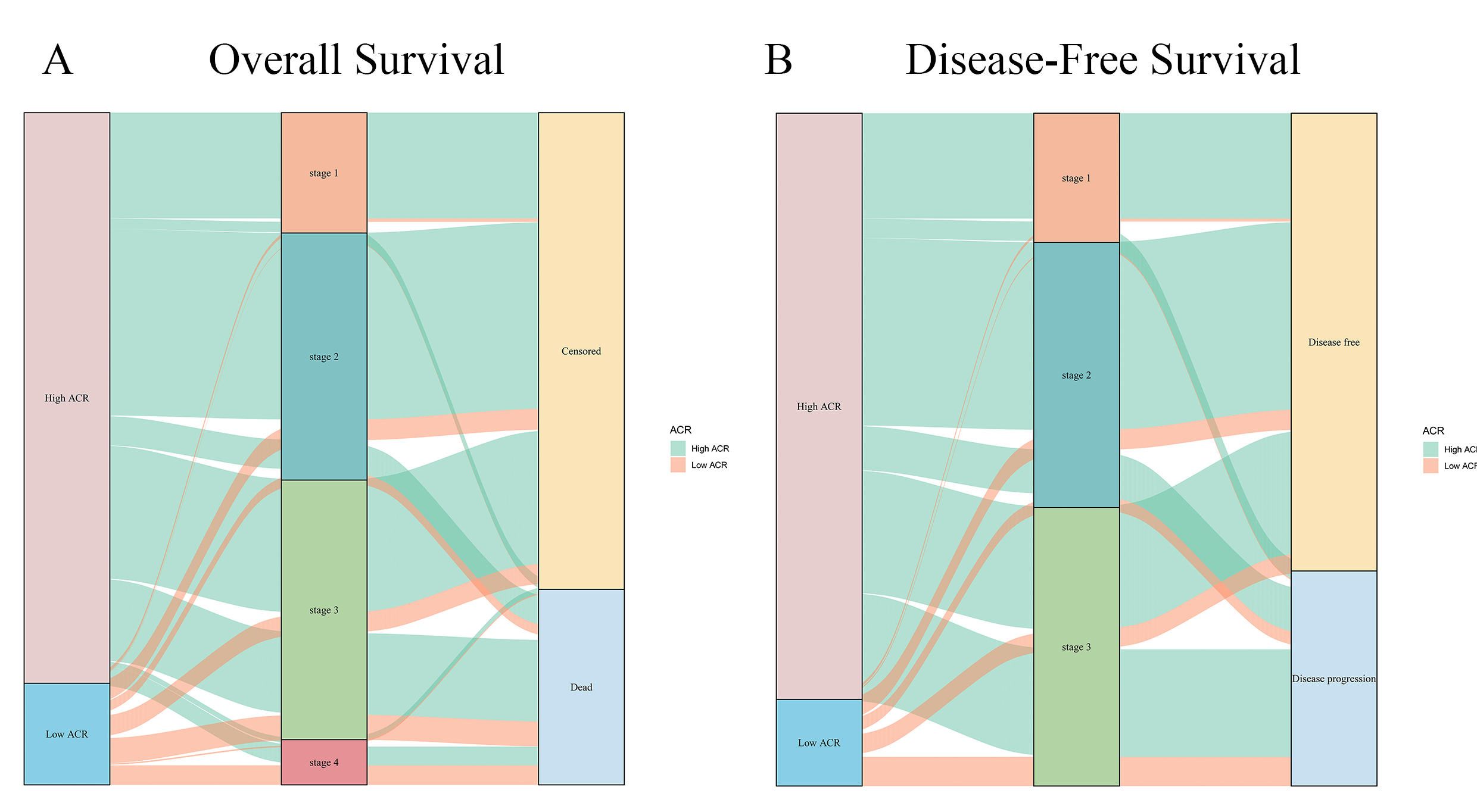


**Figure S5.** Sankey Diagram Illustrating the Relationship Between ACR Levels, TNM Staging, and Survival Outcomes (OS/DFS). (A, overall survival, B, disease-free survival). The DFS analysis only includes patients in stages Ⅰ - Ⅲ.


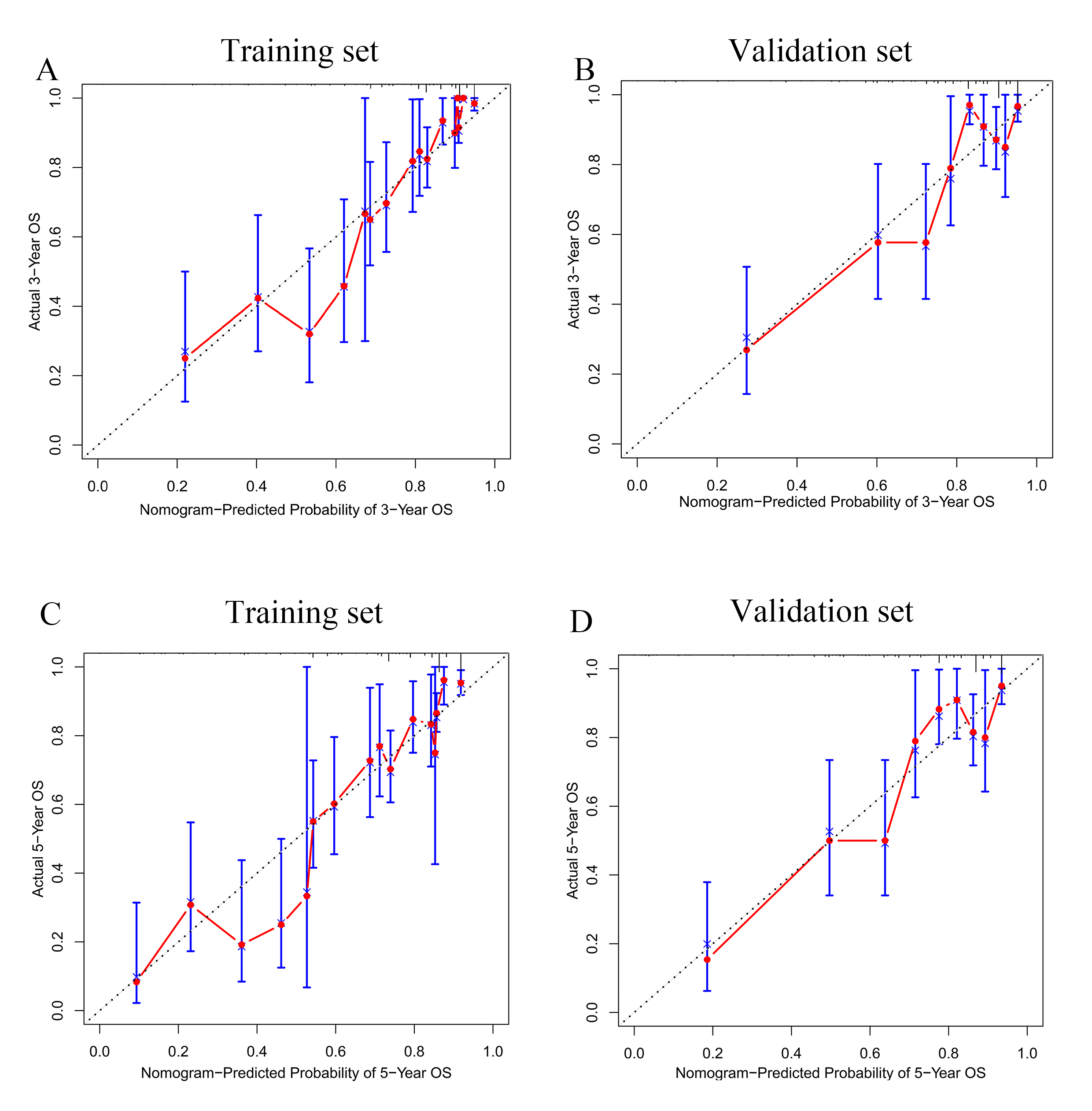


**Figure S6.** Prediction Accuracy Validation of the 3-Year (A, B) and 5-Year (C, D) OS Nomograms in Training and Validation Cohorts.


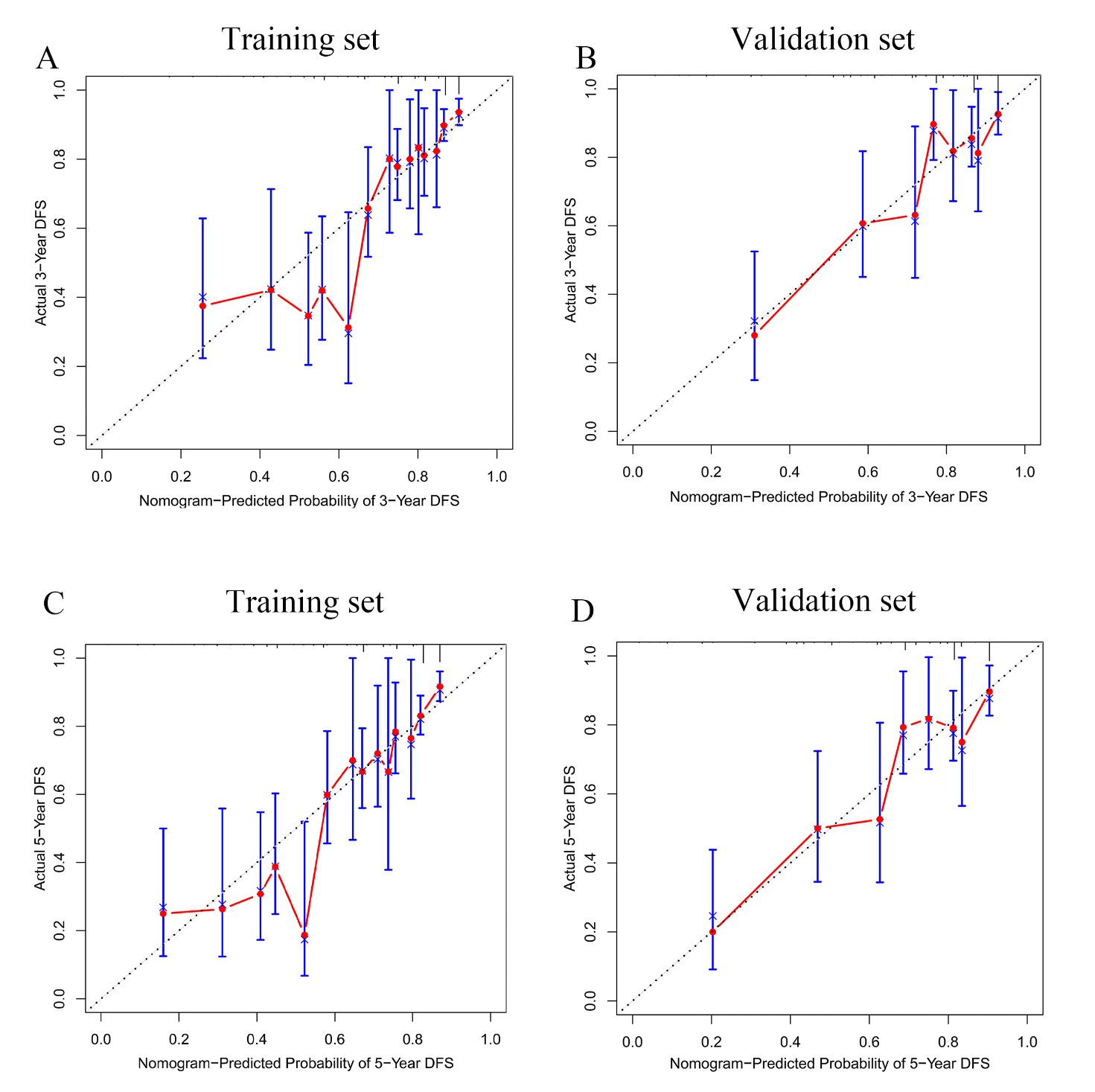


**Figure S7.** Prediction Accuracy Validation of the 3-Year (A, B) and 5-Year (C, D) DFS Nomograms in Training and Validation Cohorts.


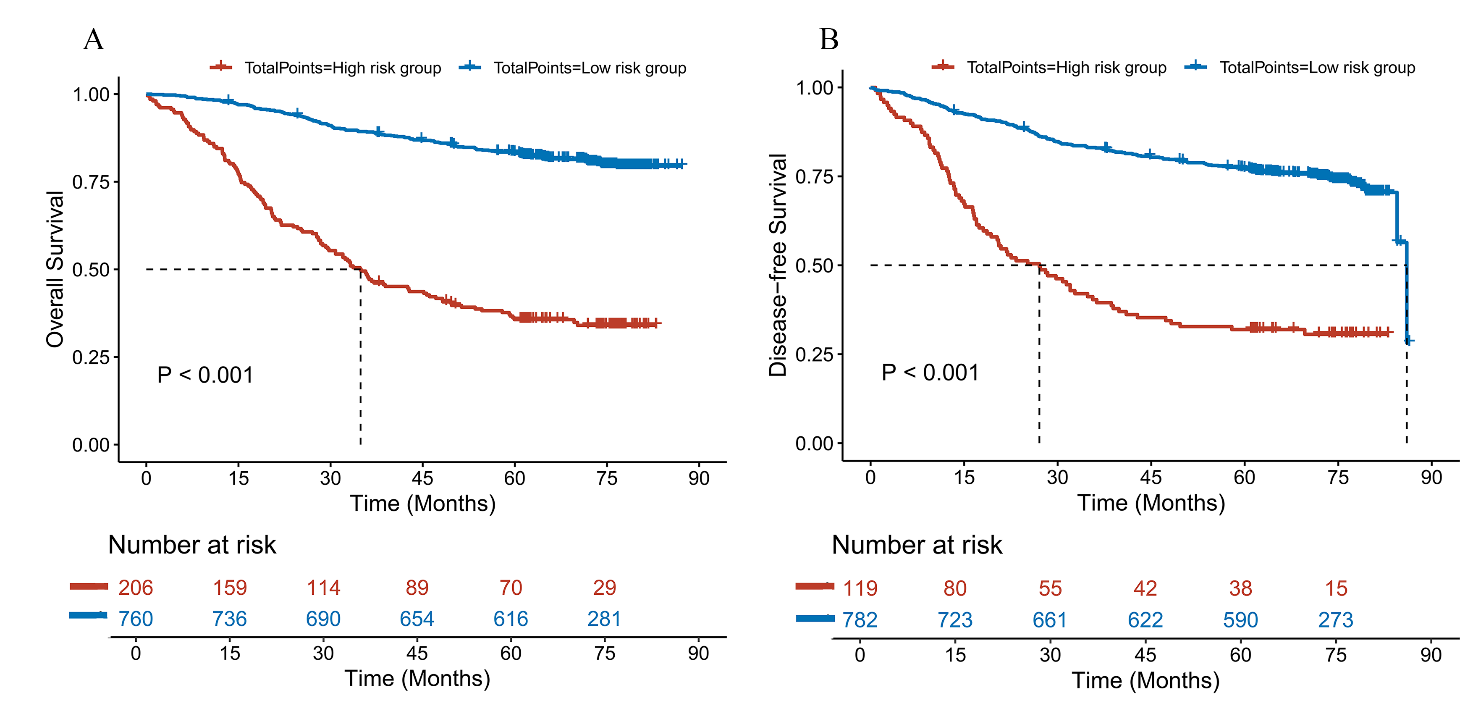


**Figure S8.** Survival analysis based on risk stratification using nomogram-derived total points. (A, overall survival, B, disease-free survival).

**Table S1** Comparison of 1-, 3-, and 5-year OS and DFS across different ACR groups

| Variable | Low ACR | High ACR |
| --- | --- | --- |
| OS |  |  |
| 1-year | 85.6% (95%CI: 80.1%-91.5%) | 97.0% (95%CI: 95.8%-98.1%) |
| 3-year | 55.5% (95%CI: 48.0%-64.2%) | 84.9% (95%CI: 82.4%-87.3%) |
| 5-year | 46.6% (95%CI: 39.1%-55.4%) | 77.8% (95%CI: 75.0%-80.7%) |
| DFS |  |  |
| 1-year | 62.3% (95%CI: 54.9%-70.7%) | 90.2% (95%CI: 88.2%-92.3%) |
| 3-year | 45.2% (95%CI: 37.8%-54.0%) | 77.0% (95%CI: 74.2%-80.0%) |
| 5-year | 39.0% (95%CI: 31.9%-47.8%) | 71.3% (95%CI: 68.2%-74.4%) |

**Table S2** Comparison of the basic characteristics between the training set and the validation set

| Characteristics | Training set  (n = 677) | Validation set  (n = 289) | χ²/Z | *P* |
| --- | --- | --- | --- | --- |
| Age (n, %) |  |  | 0.070 | 0.792 |
| ≤ 60 yr | 317 (46.8) | 138 (47.8) |  |  |
| > 60 yr | 360 (53.2) | 151 (52.2) |  |  |
| Gender (n, %) |  |  | 2.328 | 0.127 |
| Male | 374 (55.2) | 175 (60.6) |  |  |
| Female | 303 (44.8) | 114 (39.4) |  |  |
| Ileus (n, %) |  |  | 0.933 | 0.334 |
| Yes | 150 (22.2) | 56 (19.4) |  |  |
| No | 527 (77.8) | 233 (80.6) |  |  |
| Location (n, %) |  |  | 0.661 | 0.416 |
| Colon | 311 (45.9) | 141 (48.8) |  |  |
| Rectum | 366 (54.1) | 148 (51.2) |  |  |
| Histological type (n, %) |  |  | 0.014 | 0.904 |
| Ulcerative type | 569 (84.0) | 242 (83.7) |  |  |
| Non-ulcerative type | 108 (16.0) | 47 (16.3) |  |  |
| Vascular invasion (n, %) |  |  | 0.826 | 0.363 |
| Yes | 97 (14.3) | 48 (16.6) |  |  |
| No | 580 (85.7) | 241 (83.4) |  |  |
| Nerves invasion (n, %) |  |  | 0.433 | 0.511 |
| Yes | 48 (7.1) | 24 (8.3) |  |  |
| No | 629 (92.9) | 265 (91.7) |  |  |
| T stage (n, %) |  |  | 0.485 | 0.922 |
| T1 | 14 (2.0) | 7 (2.4) |  |  |
| T2 | 138 (20.4) | 54 (18.7) |  |  |
| T3 | 259 (38.3) | 111 (38.4) |  |  |
| T4 | 266 (39.3) | 117 (40.5) |  |  |
| N stage (n, %) |  |  | 1.836 | 0.399 |
| N0 | 392 (57.9) | 163 (56.4) |  |  |
| N1 | 148 (21.9) | 74 (25.6) |  |  |
| N2 | 137 (20.2) | 52 (18.0) |  |  |
| M stage (n, %) |  |  | 0.016 | 0.900 |
| M0 | 631 (93.2) | 270 (93.4) |  |  |
| M1 | 46 (6.8) | 19 (6.6) |  |  |
| TNM stage (n, %) |  |  | 0.561 | 0.905 |
| I | 125 (18.5) | 48 (16.6) |  |  |
| II | 248 (36.6) | 107 (37.0) |  |  |
| III | 258 (38.1) | 115 (39.8) |  |  |
| Ⅳ | 46 (6.8) | 19 (6.6) |  |  |
| Disease progression at last follow-up (n, %) |  |  | 0.452 | 0.501 |
| Yes | 252 (37.2) | 101 (34.9) |  |  |
| No | 425 (62.8) | 188 (65.1) |  |  |
| Status at last follow-up (n, %) |  |  | 0.615 | 0.433 |
| Dead | 202 (29.8) | 79 (27.3) |  |  |
| Censored | 475 (70.2) | 210 (72.7) |  |  |
| ALB (median, IQR) | 41.90 (5.5) | 42.00 (5.5) | -0.155 | 0.877 |
| CEA (median, IQR) | 2.15 (4.72) | 2.35 (5.01) | -0.879 | 0.379 |
| ACR (median, IQR) | 19.74 (42.73) | 17.80 (34.23) | -0.827 | 0.408 |
| ACR (n, %) |  |  | 0.276 | 0.599 |
| Low | 105 (15.5) | 41 (14.2) |  |  |
| High | 572 (84.5) | 248 (85.8) |  |  |

Table S3 The C-index of the nomogram corresponding to different cutoff values.

| Different cutoff values | C-index of nomogram | | |
| --- | --- | --- | --- |
|  | Entire dataset | Training set | Validation set |
| OS |  |  |  |
| The cutoff value is 3.5 | 0.786 | 0.805 | 0.789 |
| The cutoff value is 7.1 | 0.764 | 0.799 | 0.764 |
| The cutoff value is 3.3 | 0.776 | 0.798 | 0.774 |
| DFS |  |  |  |
| The cutoff value is 3.5 | 0.745 | 0.762 | 0.737 |
| The cutoff value is 7.1 | 0.740 | 0.758 | 0.724 |
| The cutoff value is 3.3 | 0.743 | 0.761 | 0.736 |

Table S4 C-indexes of nomograms incorporating ACR, CEA and ALB

| Nomogram | C-index for OS | C-index for DFS |
| --- | --- | --- |
| ACR-incorporated nomogram | 0.786 | 0.722 |
| CEA-incorporated nomogram | 0.780 | 0.718 |
| ALB-incorporated nomogram | 0.778 | 0.713 |

The OS nomogram incorporates the variables of age, ileus, N stage, M stage, vascular invasion, and nerve invasion, whereas the DFS nomogram includes age, N stage, vascular invasion, and nerve invasion.
